# Analysis of the Impact of Type 2 Diabetes on the Psychosocial Functioning and Quality of Life of Perimenopausal Women

**DOI:** 10.3390/ijerph17124349

**Published:** 2020-06-17

**Authors:** Małgorzata Zimny, Małgorzata Starczewska, Małgorzata Szkup, Katarzyna Karakiewicz-Krawczyk, Elżbieta Grochans, Olimpia Sipak-Szmigiel

**Affiliations:** 1 Department of Obstetrics and Pathology of Pregnancy, Pomeranian Medical University, 71-210 Szczecin, Poland; malgorzata.zimny@pum.edu.pl (M.Z.); olimpiasipak-szmigiel@wp.pl (O.S.-S.); 2 Department of Nursing, Pomeranian Medical University, 71-210 Szczecin, Poland; mlary@pum.edu.pl (M.S.); grochans@pum.edu.pl (E.G.); 3 Department of Clinical Nursing, Pomeranian Medical University, 71-210 Szczecin, Poland; katkar@pum.edu.pl

**Keywords:** body composition, type 2 diabetes, resulting symptoms, depressiveness, quality of life, perimenopausal period

## Abstract

Menopause is a natural period resulting from the decrease in hormonal activity of the ovaries. Growing hormonal deficiencies and changes in the body influence a variety of functions in women, leading to depression and decreased quality of life. The relationship between body composition, the severity of depressive and climacteric symptoms and the quality of life of women with type 2 diabetes and healthy women in the perimenopausal period was studied. Statistically significant differences were observed between the study and control groups regarding all body composition parameters except for protein and the content of torso soft tissues (*p* < 0.05). In both the study and control groups, resulting symptoms were significantly correlated with numerous body composition parameters (e.g., body mass, fat tissue mass, minerals, abdominal circumference), while symptoms of depression were significantly correlated with similar parameters only in the control group. A statistically relevant relationship was observed between the study and control groups with respect to quality of life in certain domains. The quality of life of women suffering from type 2 diabetes was worse compared with healthy women. Analysis of body composition showed significant differences between healthy women and those with type 2 diabetes. Healthy women showed a tendency to establish a link between body composition and depressiveness.

## 1. Introduction

Menopause, occurring at about the age of 50, is one of the physiological stages in the lives of women. It is a natural period resulting from the decreasing hormonal activity of the ovaries and is characterized by various somatic and psychosomatic complaints. Their severity and type vary from person to person due to genetic predispositions and external factors [1,2,3]. It is estimated that the majority of women experience menopause between the age of 45 and 55, and about 5% between the age of 40 and 45 [4].

In Poland, type 2 diabetes is a disease affecting mostly women (about 56% of all diagnosed cases), which results not so much from their greater susceptibility to the disease as from a longer life expectancy. It has also been noted that women are at a much higher risk of developing cardiovascular disease as a complication of type 2 diabetes than men (this risk is estimated to be about 50 percent higher) [5]. Due to a significant increase in the risk of developing this disease by perimenopausal women, as well as the fact that it is a growing health problem worldwide, we decided to conduct this research in a group of women in the perimenopausal period.

In the climacteric period, a significant decrease in endogenous estrogen occurs, which is accompanied by changes in body mass, adipose tissue and energy distribution; the secretion of insulin and tissue’s sensitivity to this hormone are impaired, which predisposes one to the development of type 2 diabetes [6].

In women, with age and increases in body mass, changes in adipose tissue distribution occur—gynoid fat distribution in the area of the hips and thighs is replaced with android fat distribution associated with the deposition of excessive fat tissue in the greater omentum and subcutaneous abdomen tissue [7]. The perimenopausal period predisposes women to changes in body composition in a special way. An increase in the percentage of body fat is noticeable, especially in the central body area. This is probably associated with hormonal changes, and above all an increase in androgen levels that can be caused by higher levels of luteinizing hormone or greater sensitivity to it, parallel to a reduction in the level of sex hormone binding globulin [8]. It is not yet certain whether the redistribution of adipose tissue into the abdominal cavity in peri- and postmenopausal women is more likely to be caused by involutional or menopausal changes. It has been noted, however, that it can occur even while maintaining a relatively constant body weight [9], which justifies the assessment of individual body components, and not just weight.

Many women in the perimenopausal period experience menstrual disorders; vasomotor symptoms appear, such as hot flashes, and sleep hyperhidrosis often leads to sleep disorders; and psychosomatic symptoms such as mood lability and depressiveness and also a decrease in libido and dyspareunia appear, which may negatively influence a woman’s sexual life [10]. Studies have shown that the perimenopausal period is associated with a greater risk of the occurrence of depressive symptoms. In addition, a positive relation between vasomotor symptoms and depressiveness has been established [11,12]. Other factors increasing the risk of developing depression during this period include negative life incidents, a bad attitude towards menopause, the quality of the relationship with one’s life partner, quality of life [13] and a high level of anxiety and neurotic personality [14].

Increasing hormone deficiencies and symptoms of progressing aging fundamentally influence various areas of functioning of a woman’s body, often leading to decreased self-esteem and a worse quality of life [15,16].

The aim of this study was to identify the link between body composition, the severity of depressive and climacteric symptoms and the quality of life in healthy women and those suffering from type 2 diabetes in the perimenopausal period.

## 2. Materials and Methods

All patients from the study and control groups took part in the study, which was conducted in two stages. The first part was carried out using a diagnostic poll method with standardized research tools. The inquiries were made based on standardized tools and an author’s questionnaire concerning information on sociodemographic data. The second stage involved a body composition analysis with the body analysis scale Jawon Medical IOI-353.

The following standardized research tools were used:

1. The Blatt–Kupperman Index, which is designed to objectively assess the severity of menopausal symptoms. The questionnaire includes 11 menopausal symptoms: hot flashes, perspiration, sleeping disorders, nervousness, sadness (depression), dizziness, general fatigue, arthralgia, cephalgia, palpitations and paraesthesia. The occurrence of hot flashes was assessed as 4 points multiplied by the severity scale: 0—none, 1—light, 2—medium, 3—severe (maximum 12 points). The presence of sweating, sleep disorders and nervousness was assessed as 2 points multiplied by the appropriate multiplier (0, 1, 2 or 3, maximum 6), while the remaining symptoms as 1 point multiplied by the appropriate multiplier (0, 1, 2 or 3, maximum 3). Results between 0 and 16 points were interpreted as normal, meaning lack of menopausal symptoms. Results between 17 and 25 points were interpreted as mildly severe, between 26 and 60 points as symptoms of moderate severity and results above 30 points indicated great severity of symptoms [17].
2. The Beck Depression Inventory is a self-report inventory of mood and depressiveness. It consists of 21 multiple-choice questions. Every question has four possible answers, each of which is assessed differently. The following thresholds have been accepted: 0–11 points—no depression, 12–26 points—mild depressive episode, 27–49 points—moderate depressive episode and 50–63 points—severe depressive episode [18].
3. The Short-Form-36 Health Survey (SF-36) was used to assess the quality of life of the surveyed women. The questionnaire allows us to assess everyday functioning considering two areas—physical and mental—in 11 domains: physical functioning—P.F., role limitations due to physical problems—R.P., bodily pain—B.P., general health perception—G.H., vitality—V.T., social functioning—S.F., mental health—M.H., role limitation due to emotional problems—R.E, health transition—H.T., physical component summary—P.C.S. and mental component summary—M.CS. The quality of life in each domain is expressed with a number ranging from 0 to 100. The greater the number, the better the quality of life [19].

### 2.1. Procedures of the Body Composition Assessment

The measuring test was carried out using body analysis scale Jawon Medical IOI-353. The Aim Of This Test Was To Collect Anthropometric Data And Data On Visceral Tissue. The Body’s Individual Components, Basal Metabolic Rate And Age Matched Of Body Were Evaluated Using The Analyzer. The Device Uses Bioelectrical Impedance Analysis (B.I.A.).

The measuring test was carried out using a body analysis scale, non-invasive and effective way of studying individual components in the human body. The following parameters were determined during the examination:

- Body weight (kg); height (cm); L.B.M.—lean body mass (kg); S.L.M.—soft lean mass (kg); T.B.W.—total body water (%); P.B.F.—percent of body fat (%); M.B.F.—mass of body fat (kg); B.M.I.—body mass index (kg/m2); fatness [%]; V.F.A.—visceral fat area (cm2); A.C.—abdominal circumference (cm); W.H.R.—waist-hip ratio (cm); B.M.R.—basal metabolic rate (kcal); T.E.E.—total energy expenditure (kcal); A.M.B.—age matched of body (years); determination of silhouette type (9 silhouette types); protein content (kg); mineral content (kg); impedance (Ω); fat content in the individual segments of the body: lower extremities, upper extremities and torso; soft tissue content in the individual segments of the body: lower extremities, upper extremities and torso; determination of calorie intake in recommended diet; assessment of suggested amount of calories which need to be burned throughout physical activity; aim until control (kg) and duration of therapy (weeks). The basal metabolic rate was calculated based on the results of body composition, age, weight and the Impedance Index.
- The body analysis scale Jawon Medical IOI-353 has an EC0197 certificate and meets the requirements of the MDD 93/42EEC directive regarding medical devices. As recommended by the producer, Jawon Medical IOI-353 must not be used in pregnant patients and those with a pacemaker. All study participants were informed about the contraindications to perform body analysis and confirmed their absence. The study was conducted in the morning; all patients had at least an 8-h fasting period.

### 2.2. Statistical Analysis

Analysis of quantitative variables was conducted using basic descriptive statistics: average, standard deviation, median, quartiles, minimum and maximum. Analysis of qualitative variables was carried out by calculating the number and percent of occurrences of each value. Inferential statistics were conducted based on an assumption of normal distribution and the following statistical tests. A comparison of qualitative variable values in the groups was performed using a chi-squared test (with Yates’ correction for 2 × 2 tables) or Fisher’s exact test in tables in which expected numbers were low. A comparison of quantitative variables in the two groups was conducted using the Mann–Whitney test (when one of the variables did not have a normal distribution). The normality of the distribution was checked with the Shapiro–Wilk test. During analysis, 0.05 was adopted as a significance level—all *p* values below 0.05 were interpreted as statistically significant.

Moreover, a correlation coefficient informing about the degree of correlation between analyzed variables was used. Correlation between two quantitative variables was analyzed using Spearman’s rank correlation coefficient (when at least one of the variables did not have a normal distribution).

### 2.3. Organization and Course of Study

All subjects gave their informed consent for inclusion before they participated in the study. The study was conducted in accordance with the Declaration of Helsinki, and the protocol was approved by the Bioethical Commission of the Pomeranian Medical University (KB-0012/90/15, 22 June 2015).

The inclusion criteria for the study were diagnosed type 2 diabetes; no thyroid, mental or cancerous diseases at the time of the study or in the patient’s history as well as no history of bariatric surgery.

The final study was conducted among women from West Pomeranian Voivodeship. The study group included patients aged between 45 and 65 years with clinically diagnosed type 2 diabetes, which was confirmed by a general practitioner. The control group consisted of healthy women aged between 45 and 65 years who had not been diagnosed with type 2 diabetes, based on a clinical interview and fasting glucose test; no present or past thyroid, mental or cancerous diseases and no history of bariatric surgery. The patients found out about the study from leaflets and posters placed in public spaces such as town halls, schools, national health care institutions, diabetes outpatient clinics and hospital wards.

Each person both from the study and control group who met the inclusion criteria received background information regarding the study: topic, aim, study characteristics and tips on filling out the questionnaire. After giving written consent, patients received questionnaire forms. Thereafter, they were subjected to a body composition analysis with a Jawon Medical IOI-353 analyzer. The whole procedure was performed by trained and qualified staff. Each patient received a printout from the analyzer with its interpretation. Respondents were informed that participation in the study was both voluntary and anonymous and that obtained results would be used for research purposes and would not influence their therapy. Additionally, the participants were informed about the possibility to resign at any stage of the study without having to justify their decision.

### 2.4. Characteristics of the Study and Control Groups

The study included 172 women in the perimenopausal period aged between 45 and 65. The study group included 68 women with diagnosed type 2 diabetes, and 104 healthy participants constituted the control group. All the respondents lived in West Pomeranian Voivodeship.

The average age of respondents was 54.9 years. In the study group (women with type 2 diabetes), the average age was 58.7, while the average age in the control group was 52.3.

More than half of participants (56.4%) lived in a city with a population greater than 100 thousand. The majority had secondary (45.3%) or higher education (33.7%). Most women from the study group had secondary education (53.9%). In the control group, however, most women had higher education (45.2%). The vast majority of those surveyed were married (76.2%). Most women were professionally active (75.0%).

A significant majority of women had not been menstruating (74.4%). Among those surveyed who did not menstruate, an average period from their last menstruation was 9.79 years. The time that had passed since the last menstruation was longer in the study group and reached 11.88 years on average, with 7.8 years in the control group. The majority of women who were still menstruating had their menstruations regularly (74.4%).

The mean arterial pressure in the study group was 135/76 mmHg, and in the control group, it was 114/69 mmHg―the differences in the values of both diastolic and systolic pressure were statistically significant. The mean systolic pressure in the study group was 135 mmHg, and the control group was 114 mmHg. The mean diastolic pressure in the study group was 76 mmHg, and the control group was 69 mmHg.

In the study group, the average duration of illness was 8.4 years; the duration of type 2 diabetes was counted from its diagnosis. The vast majority of women (75%) used oral medications in the treatment of diabetes, and the other patients were treated with insulin—16.18%, diet and exercise—7.35% and 1.47% used an insulin pump. Based on the self-assessment of health status, the examined women did not report symptoms of kidney damage—86.76%, diabetic foot—88.24%, thrombosis—83.82%, ischemic disease of the lower limbs—82.35% and cerebrovascular diseases—94.12%. The most often mentioned complications were mild and moderate visual disturbances—64.71% and very severe visual disturbances—4.41%, while 30.88% of women did not observe this complication (Table 1).

## 3. Results

The assessment of the severity of menopausal symptoms according to the Blatt–Kupperman scale was great in 7.0%, average in 8.1% and mild in 23.8% of the surveyed, and 61.1% of respondents did not manifest any symptoms. No statistically significant differences between the study and control groups were observed (*p* > 0.05).

Thereafter, the mental functioning of patients was assessed. For this purpose, the Beck Depression Inventory was used. Combined analysis of all surveyed women showed that the majority did not present symptoms of depression (77.9%). However, 15.1% showed mild, 4.7% presented with average and 2.3% with severe symptoms of depression. No statistically significant differences were observed between the study and control group as far as depressiveness was concerned (*p* > 0.05).

Statistically significant differences between the study and control groups were observed regarding the quality of life in certain domains. In the study group, the quality of life was lower in the following domains: physical functioning (P.F.), role limitations due to physical problems (R.P.), bodily pain (B.P.), general health perception (G.H.) and physical functioning, or physical component summary (P.C.S.). The greatest differences between the study and control group were observed within R.P. and R.E. (role limitation due to emotional problems). The most similar results for both groups were obtained in the case of M.C.S.—mental component summary, or mental functioning (Table 2).

The vast majority of surveyed women presented an obese body type (74.4%); high body fat was noted in 13.4% of women, 9.9% had a normal physique and other respondents presented with other types of body shapes (2.3%). A statistically significant difference between the study and control group regarding silhouette type was observed (*p* < 0.001). Patients in the study group more frequently were obese (92.6%) and less frequently had a normal body type (4.4%) or with high fat percentage (1.5%). In the control group, 62.5% of women were obese, 13.5% had a normal body structure and 21.1% had a high percentage of body fat.

Statistically relevant differences, with significance level *p* < 0.05, were observed between the study and control group in almost every measured parameter with the exception of protein and torso soft tissue mass. In the study group, measured parameters were higher, especially body mass and fat tissue mass. Analysis of the abdominal area in study group respondents showed standard body mass was lower, but all the other parameters were lower in the control group. The greatest difference in fat tissue distribution between both groups considered the torso and both extremities. Statistically significant differences between the study and control group with a statistical significance level *p* < 0.05 were also noticed as far as the contents of controls and recommendation guides were considered. In the study group, impedance (the measure of the opposition of the body’s tissues) was lower. However, metabolic age, aim (number of kilograms that needed to be reduced) and therapeutic period (time during which the patient should achieve the goal weight) were higher in the control group (Table 3).

In the study group, menopause symptoms were significantly correlated with 12 parameters (among others, mass of the body and fat tissue, percentage of total body water, estimated abdominal circumference measured around the umbilicus, content of fat tissue in the extremities) and in the control group, with 16 parameters (such as mass of the body and fat tissue, percentage of body fat, B.M.I., visceral fat area, estimated umbilical circumference measured around the umbilicus, content of fat tissue in the extremities), and it was a weak positive correlation. Symptoms of depressiveness did not significantly correlate with any of the measured parameters in the study group. In the control group, on the other hand, symptoms of depressiveness were correlated with 11 parameters (including mass of the body and fat tissue, estimated abdominal circumference around the umbilicus, content of fat tissue in extremities)—it was a weak positive relationship (Table 4).

In the study group, the domain physical functioning (P.F.) was significantly correlated with tissue mass in the left arm and metabolic age, and in the control group, it was only correlated with metabolic age. All those links were negative. Similarly, the quality of life in the domain of physical restrictions in the study group was correlated with 19 parameters (such as body and fat tissue mass, total body water and fat tissue percentage, B.M.I., visceral fat area, estimated abdominal circumference around the umbilicus and hip-to-waist ratio). In addition, it was a weak negative link. In the control group, there was no correlation between any of the factors (Table 5).

In the study group, the bodily pain (B.P.) domain was correlated with 11 parameters, and in the control group, no significant relationship existed. Those correlations were weakly negative. Only the link between impedance was weakly positive (Table 6).

In the study group, quality of life in the mental health (M.H.) domain was significantly correlated with body mass, minerals and content of fat tissue in the left leg. In comparison, it did not correlate with any of the parameters in the control group. Those relationships were negative (Table 6).

The quality of life in the physical component summary (P.C.S.) domain was correlated relevantly with 12 parameters, and in the control group, it did not correlate with any. Those relationships were weakly negative (Table 7).

The results of the body composition analysis carried out with the Jawon Medical IOI-353 device did not significantly correlate with the quality of life in the remaining domains, i.e., general health perception, vitality, social functioning, role limitations due to emotional problems, health transition and mental component summary (*p* > 0.05).

## 4. Discussion

The perimenopausal period is a time when symptoms related with hormonal changes reveal themselves. Besides menopausal and psychological symptoms, we can also observe weight gain. It is estimated that 40% of women after menopause are overweight and more than 20% are obese [20,21]. In our study, 74.4% of surveyed women in both groups had excessive weight or obesity, while significantly more women were obese in the group of patients with diagnosed type 2 diabetes. It is estimated that the percentage of obese women in Poland doubles after menopause, and visceral fat tissue increases over 50% [22]. This phenomenon has been observed in women worldwide—excessive weight and obesity has been observed, among others, in 60% of American and Spanish women over 40 and in 76% of Brazilians [23,24].

Excessive weight and obesity are a problem of patients with type 2 diabetes, which was confirmed with our own studies. Nationwide, the ARETAEUS1 study showed that patients with newly diagnosed type 2 diabetes were overweight (37.4%) and obese (51.9%) [25]. Studies have shown that visceral fat tissue mass and visceral obesity are significantly correlated with insulin resistance [26,27].

According to Jones and Sutton’s studies, the higher a woman’s body mass is (B.M.I. > 30 kg/m^2^), the worse is their biopsychosocial functioning [28]. Similar results indicating that body appearance influences both the severity of menopausal symptoms and mental wellbeing were achieved by Stadnicka and Iwanowicz-Paulus [29].

A decreasing level of estrogen in the perimenopausal period may influence adipocytes’ biology, resulting in increased deposition of fat tissue in the abdominal region, which is also caused by a higher androgen to estradiol ratio [30,31]. One of the main complications of obesity, especially the visceral type, is cardiovascular disease [32]. Douglas and Ginsburg, paying attention to adipose tissue distribution, showed a significant link between waist-to-hip ratio (W.H.R.) and incidence of cardiovascular system diseases [33]. Similarly, longitudinal studies have suggested a relevant relationship between obesity, especially the visceral type, and an increased risk of developing atrial hypertension, arteriosclerosis and coronary artery disease [34,35].

In Toth et al.’s studies, it was established that postmenopausal women had 36% more of visceral adipose tissue and a 49% greater area of visceral fat than premenopausal women [36]. In addition, in Pachocka’s studies, visceral obesity dominated, especially among women in the perimenopausal period. However, no statistically significant differences in overall body fat were observed [37].

Metanalysis of cross-sectional studies carried out by Ambikairajah’s team indicated an increase in central adipose tissue mass and decreased percentage of adipose tissue in lower extremities among postmenopausal women [31]. In our own studies, there was a statistically significant difference regarding all measured parameters constituting analysis of the abdominal region. It was observed that women with type 2 diabetes had visceral adipose tissue and an increased estimated abdominal circumference around the umbilicus.

Major increases in the prevalence of those overweight and obesity generates both social and health problems, especially during the perimenopausal period, and has a significant influence on the quality of a woman’s life [38,39,40], which should be considered in different aspects, from subjective self-image, the characteristic physical state at a given moment, to perspective of specific perimenopausal period physiological changes and their consequences [41].

In the evaluation of women’s quality of life, Jurczak et al. established that in women in the perimenopausal period, among all domains assessed their physical and mental functioning and physical fitness as the best, and vitality and mental health as the worst [42]. In contrast, Kolarzyk et al. showed that women in the perimenopausal period who had higher education, lived in cities and were professionally active assessed their physical and mental sphere as the best, and the women with primary or secondary education living in the country assessed both of those spheres as the worst [43]. Our own studies showed that women functioned better in the physical than mental aspect. Women with type 2 diabetes had a lower quality of life, especially in domains related with restriction of activity due to health condition and caused by emotional problems. Better quality of life in the aspect of physical prowess promoted lower values of metabolic age and mass of soft tissues in the left arm. The reduction of the majority of measured parameters was influenced by the quality of life in the aspect of role limitations due to physical problems, physical functioning and bodily pain. Additionally, it has been proven that the lower the levels of parameters such as body mass, minerals and content of adipose tissue in the left leg, the higher is the patient’s quality of life.

In Jakubca et al.’s studies, a positive correlation between body composition and quality of life in women with gynoid fat distribution was established, while a negative relationship in women with android fat distribution was noticed. In other words, women from the first group were characterized by a higher quality of life than women from the second group [44].

The type and severity of menopausal symptoms varies from person to person. In Genazzani et al.’s study, 94% of women suffered from menopausal symptoms [45]; in Orzechowska et al.’s study, more than 80% [46] and in Dmoch-Gajzlerska et al.’s studies, all of the surveyed women suffered from menopausal symptoms but they varied in intensity [47]. Conversely, in studies of Wieder-Huszla et al., the majority of women (68.2%) did not experience menopausal symptoms [48]. Our own studies showed a lack of symptoms in the majority of women. No statistically significant relationship (*p* > 0.05) between groups was observed; however, women with average or severe symptoms were more numerous in the group of women with type 2 diabetes than in the control group. In women with type 2 diabetes, menopausal symptoms significantly correlated with body mass; mass of adipose tissue; total body water percentage; estimated abdominal circumference at the umbilicus; distribution of adipose tissue in the upper left limb, trunk and lower right limb; distribution of soft tissues in the upper right limb; aim to control and duration of therapy.

Depressive disorders are a common occurrence in the perimenopausal period. A typical presentation is tearfulness, irritability, emotional lability and poor concentration. The number and severity of depressive symptoms increases with age [49]. Studies of Pol Senior showed that one in five people aged between 55 and 59 years and almost 30% of people above the age of 65 experienced depressive symptoms, and women suffered more frequently [50]. On the contrary, studies carried out by Barnaś et al. showed that 61% of surveyed women in the perimenopausal period were not depressed [51]. Our own study also showed that the vast majority of respondents (77.9%) did not present with depressive symptoms. Our results did not indicate the presence of a statistically significant difference in the incidence and severity of depression between the group of women with type 2 diabetes and the control group. This is quite surprising in light of the results obtained by other authors indicating a reciprocal relationship between depression and type 2 diabetes―depression increases the risk of developing diabetes, while the diagnosis of diabetes is often associated with an increased risk of depression and may lead to a more severe course of this disease. It is estimated that depression occurs in about a quarter of people with depression [52], which is consistent with the results obtained in our study. On the other hand, the study of 815 Polish perimenopausal women without type 2 diabetes showed that 25.5% of them had depressive symptoms [14]. In light of these reports, the conclusion is drawn that menopausal status and related changes have a greater impact on the occurrence of depression in middle-aged women than type 2 diabetes alone. This conclusion should be treated as preliminary and requiring further verification.

Walczak and Wiśniewska established that symptoms of depression increase in severity when impairment of cognitive functions is escalating, which is certainly associated with less effective functioning [53]. Other researchers have claimed that vasomotor symptoms of menopause play a role in the prevalence of depression [54].

Surprisingly, the results of our study showed that body composition had no influence on depressive symptoms in women with type 2 diabetes. Inferences about the reasons for this situation is beyond the scope of our study, but our attention was also drawn to the low percentage of people with diabetes whose treatment was based only on diet and exercise (only 7.35%), without including pharmacological treatment. Perhaps in the study group, there was a long-lasting tendency to lead a low-active lifestyle and not attach special importance to diet, which in turn contributed to the onset of type 2 diabetes, but did not have a major impact on the development of depression. This issue seems intriguing but requires further research to find the causes of this phenomenon. In the control group, there was a link between body composition, its individual components and severity of depressive symptoms. The more serious were the depressive symptoms experienced by the patient, the greater were the values of measured parameters. Studies have shown that a relevant relationship between the prevalence of depression and an increase of abdominal adipose tissue [55] and a high Body Mass Index [56] exists. Weber-Hamann et al., in their studies, noticed an increased abdominal tissue volume in patients with depression and increased levels of cortisol [57]. A patten has proven that the better the functioning in the physical domain of the quality of life, the fewer the symptoms of depression that occur [58].

Type 2 diabetes is a chronic disease resulting in a number of complications, including life-threatening cardiovascular problems, the risk of which is 2–4 times higher in people with diabetes than in the general population. It is estimated that in 2030, around 2.5 million diabetics will live in the world, and the highest prevalence will be in the group of middle-aged people (45–64 years old) [59]. Considering the importance of the type 2 diabetes problem for public health, it seems reasonable to conduct research to analyze the impact of this disorder on psychosocial functioning and quality of life in various groups of the population, especially those predisposed to this disease.

### Limitations

In order to achieve unbiased data, a standardized research tool and a professional body composition analyzer were used. Increasing the number of participants, especially the ones with diagnosed type 2 diabetes, is a chance to increase the credibility of carried out studies. Moreover, differences in average age between both groups (58.7 vs. 52.3) may be a potentially distorting factor. Achieved results, however promising, need to be replicated in order to be applied to a wider population.

## 5. Conclusions

Even though symptoms of depression and menopause were experienced at a similar degree in both groups, the quality of life of women suffering from type 2 diabetes was worse in comparison with healthy women.

The application of a reliable and efficient method of assessment of body composition indicates considerable variation between healthy women and women suffering from type 2 diabetes who presented with more body fat.

Healthy women showed a tendency to establish a relationship between body composition (especially with components related to greater fat content in the body) and depressiveness, which was absent in patients with type 2 diabetes.

The quality of life in the aspects regarding physical functioning show a correlation with body composition but only among women suffering from type 2 diabetes. No such link has been confirmed in healthy patients.

## Figures and Tables

**Table 1:** Characteristics of the group of women with type 2 diabetes with regard to the treatment methods and comorbidities.

Treatment			
Methods	*n* = 68	%	Average Time of Use (Years)
Only Diet and Physical Effort	5	7.35	4.3
Oral Agents	51	75	7.8
Insulin	11	16.18	5.6
Insulin Pump	1	1.47	4.0
**Comorbidities**			
**The Level of Severity**	**None *n* (%)**	**Mild or Moderate *n* (%)**	**Severe *n* (%)**
Kidney Damage	59 (86.76)	9 (13.24)	0 (0)
Diabetic Foot	60 (88.24)	7 (10.29)	1 (1.47)
Thrombosis	57 (83.82)	10 (14.71)	1 (1.47)
Ischemic Disease of the Lower Limbs	56 (82.35)	11 (16.18)	1 (1.47)
Cerebrovascular Disease	64 (94.12)	4 (5.88)	0
Visual Disturbances	21 (30.88)	44 (64.71)	3 (4.41)

**Table 2:** Characteristics of the quality of life in different domains in the study and control groups.

Domain	Group	*n*	M ± S.D.	Me	Min–Max	Q_1_–Q_3_	*p*
Physical Functioning—P.F.	Study	65	67.2 ± 25.8	75	0–100	50–90	<0.001
	Control	100	82.3 ± 16.9	90	20–100	75–95	
Role Limitations due to Physical Problems—R.P.	Study	68	62.1 ± 42.9	75	0–100	18.8–100	0.005
	Control	102	80.9 ± 30.9	100	0–100	75–100	
Bodily Pain—B.P.	Study	67	57.8 ± 25.4	60	10–100	40–80	0.02
	Control	102	67.1 ± 21.5	70	10–100	50–80	
General Health Perception—G.H.	Study	66	39.3 ± 12.7	40	0–70	30–50	<0.001
	Control	102	48.6 ± 12.1	50	20–85	40–55	
Vitality—V.T.	Study	65	55.4 ± 18.6	60	0–100	40–70	0.439
	Control	102	58.1 ± 19.0	60	5–95	45–70	
Social Functioning—S.F.	Study	67	69.0 ± 27.8	62.5	0–100	50–100	0.051
	Control	100	78.1 ± 22.4	87.5	12.5–100	62.5–100	
Role Limitation Due to Emotional Problems—R.E.	Study	68	69.1 ± 42.8	100	0–100	33.3–100	0.077
	Control	102	81.1 ± 34.1	100	0–100	66.7–100	
Mental Health—M.H.	Study	59	64.8 ± 16.1	60	36–100	52–76	0.365
	Control	102	65.8 ± 13.0	64	36–100	60–76	
Health Transition—H.T.	Study	68	41.9 ± 25.0	50	0–100	25–50	0.141
	Control	102	47.6 ± 20.1	50	0–100	50–50	
Physical Component Summary—P.C.S.	Study	62	55.7 ± 18.7	60.38	7.5–83	43.4–69.3	<0.001
	Control	100	68.3 ± 14.3	71.7	18.9–88.7	60.4–79.3	
Mental Component Summary—M.C.S.	Study	55	61.9 ± 16.2	62.5	25–92.9	50.9–73.2	0.202
	Control	100	65.6 ± 14.1	66.07	30.4–96.4	56.7–76.8	

Mann–Whitney’s Test, *n*—number, M—mean, S.D.—standard deviation, Me—median, Q1—first quartile, Q3—third quartile, *p*—level of significance.

**Table 3:** Characteristics of the study and control group considering body composition analysis, control guides and recommendations.

Parameter	Group	*n*	M ± S.D.	Me	Min–Max	Q_1_–Q_3_	*p*
Body Mass (kg)	Study	68	82.4 ± 14.9	81.3	56.7–122.6	69.9–92.5	<0.001
	Control	104	72.1 ± 14.5	71.3	16.3–114.1	62.7–80	
L.B.M. (kg)	Study	68	49.6 ± 7.0	48.9	37.5–64.5	43.8–54.3	0.009
	Control	104	46.8 ± 5.8	46.5	35.9–70.1	42.9–50.1	
Fat Tissue Mass (kg)	Study	68	32.8 ± 9.0	32.2	15.5–58.1	25.9–39.3	<0.001
	Control	104	25.9 ± 8.6	24.5	6–57.5	20.2–30.6	
S.L.M. (kg)	Study	68	45.1 ± 6.4	44.3	33.8–58.9	40.1–49.6	0.018
	Control	104	42.7 ± 5.2	42.5	33.3–63.7	39.2–45.6	
Minerals (kg)	Study	68	4.6 ± 0.8	4.6	3.3–6.8	4–5.2	<0.001
	Control	104	4.1 ± 0.7	4	2.6–6.4	3.6–4.5	
Protein (kg)	Study	68	9.7 ± 3.8	9.1	6.8–38.9	8.3–10.1	0.197
	Control	104	9.0 ± 1.0	9.0	7.2–13.2	8.3–9.5	
T.B.W. (kg)	Study	68	35.3 ± 5.9	35.1	11.1–46.4	31.5–39.2	0.019
	Control	104	33.7 ± 4.2	33.5	25.8–50.5	30.9–36.0	
P.B.F. (%)	Study	68	39.2 ± 5.0	40.3	22.3–47.6	36.7–42.5	<0.001
	Control	104	34.9 ± 5.9	35.3	10.2–51.5	32.0–38.9	
B.M.I. (kg/m^2^)	Study	68	32.7 ± 5.9	32.6	20.5–50	28.6–35.9	<0.001
	Control	104	27.8 ± 5.6	26.9	17.7–53.7	24.2–30.8	
Level (steps)	Study	68	15.2 ± 3.4	16	3–20	14–17	<0.001
	Control	104	12.2 ± 3.9	12	1–20	10–15	
V.F.A. (cm^2^)	Study	68	159.9 ± 63.7	156.5	30–316	117–204.8	<0.001
	Control	104	111.1 ± 59.5	101	20–386	75–135	
A.C. (cm)	Study	68	94.5 ± 10.6	93.8	74.1–124.3	86.4–102.1	<0.001
	Control	104	86.6 ± 10.4	84.7	62.9–123.6	79.7–91.9	
W.H.R.	Study	68	0.9 ± 0.1	0.9	0.7–11	0.9–1.0	<0.001
	Control	104	0.8 ± 0.1	0.8	0.8–1.1	0.8–0.9	
Std. Mc. (kg)	Study	68	55.0 ± 7.7	54.9	11.1–77.7	51.8–58.1	<0.001
	Control	102	58.1 ± 4.7	58.3	42.4–71.1	55.2–61.1	
L. Arm—Adipose Tissue (kg)	Study	68	2.1 ± 0.6	2.1	1.0–3.6	1.7–2.6	<0.001
	Control	104	1.7 ± 0.5	1.6	0.4–3.6	1.3–2.0	
L. Arm—Soft Tissues (kg)	Study	68	3.0 ± 0.8	2.8	2.0–8.5	2.6–3.2	0.018
	Control	104	2.7 ± 0.4	2.7	2.1–4.4	2.5–3.0	
R. Arm—Dipose Tissue (kg)	Study	68	2.2 ± 0.6	2.1	1.0–4.2	1.8–2.6	<0.001
	Control	104	1.7 ± 0.6	1.6	0.4–3.5	1.3–2.0	
R. Arm—Soft Tissues (kg)	Study	68	2.9 ± 0.5	2.8	1.9–4.0	2.6–3.2	0.042
	Control	104	2.7 ± 0.4	2.7	2.1–4.4	2.5–3.0	
Trunk—Adipose Tissue (kg)	Study	68	16.8 ± 4.7	16.5	7.0–29.9	13.3–20.2	<0.001
	Control	104	13.3 ± 4.4	12.5	3.1–29.6	10.3–15.7	
Trunk—Soft Tissues (kg)	Study	68	22.4 ± 2.9	22.2	17.4–30.6	20.5–24.5	0.06
	Control	104	21.5 ± 2.3	21.4	16.8–29.6	19.8–22.8	
L. Leg—Adipose Tissue (kg)	Study	68	5.8 ± 1.7	5.8	1.8–10.5	4.6–7.1	<0.001
	Control	104	4.7 ± 1.6	4.4	1.1–10.4	3.6–5.5	
L. Leg—Soft Tissues (kg)	Study	68	8.4 ± 1.5	8.2	6.1–14.1	7.2–9.2	0.017
	Control	104	7.8 ± 1.1	7.7	5.9–12.4	7.1–8.5	
R. Leg—Adipose Tissue (kg)	Study	68	5.9 ± 1.6	5.8	2.7–10.5	4.7–7.0	<0.001
	Control	104	4.7 ± 1.5	4.4	1–10.4	3.6–5.5	
R. Leg—Soft Tissues (kg)	Study	68	8.3 ± 1.3	8.2	6.1–11.2	7.3–9.1	0.013
	Control	104	7.9 ± 1.1	7.7	6.0–13.0	7.1–8.5	

Mann–Whitney’s Test, L.B.M.—lean body mass (kg); S.L.M.—soft lean mass (kg); T.B.W.—total body water (%); P.B.F.—percent of body fat (%); B.M.I.—Body Mass Index (kg/m^2^); V.F.A.—visceral fat area (cm^2^); A.C.—abdominal circumference (cm); W.H.R.—waist hip ratio; Std. Mc.—standard body mass (kg); *p*—statistical significance level.

**Table 4:** Correlation of body composition with severity of menopausal and depressiveness symptoms in the women from the study and control groups.

Parameters	Severity of Menopausal Symptoms				Severity of Depressiveness			
	Study Group		Control Group		Study Group		Control Group	
	Correlation Coefficient	*p*	Correlation Coefficient	*p*	Correlation Coefficient	*p*	Correlation Coefficient	*p*
Body Mass (kg)	0.29	0.016 *	0.221	0.024 *	0.167	0.174	0.193	0.05 *
L.B.M. (kg)	0.226	0.064	0.103	0.296	0.087	0.483	0.16	0.105
Fat Tissue Mass (kg)	0.267	0.028 *	0.236	0.016 *	0.151	0.22	0.221	0.024 *
S.L.M. (kg)	0.213	0.082	0.087	0.377	0.075	0.543	0.148	0.134
Minerals (kg)	0.278	0.022 *	0.198	0.044 *	0.157	0.202	0.213	0.03 *
Protein (kg)	0.157	0.202	0.036	0.72	0.04	0.749	0.092	0.354
T.B.W. (kg)	0.28	0.021 *	0.101	0.306	0.126	0.304	0.158	0.109
P.B.F. (%)	0.193	0.114	0.213	0.03 *	0.131	0.289	0.179	0.069
B.M.I. (kg/m^2^)	0.203	0.096	0.224	0.023 *	0.122	0.32	0.177	0.072
Fatness (%)	−0.086	0.656			−0.109	0.573		
Level (steps)	0.184	0.133	0.213	0.03 *	0.133	0.279	0.176	0.074
V.F.A. (cm^2^)	0.195	0.11	0.22	0.025 *	0.125	0.311	0.182	0.064
A.C. (cm)	0.266	0.028 *	0.236	0.016 *	0.151	0.22	0.22	0.025 *
W.H.R.	0.199	0.104	0.207	0.035 *	0.121	0.327	0.188	0.056
Std. Mc. (kg)	0.098	0.429	−0.049	0.627	0.05	0.687	0.034	0.735
L. Arm—Adipose Tissue (kg)	0.254	0.037 *	0.236	0.016 *	0.105	0.395	0.212	0.031 *
L. Arm—Soft Tissues (kg)	0.205	0.094	0.057	0.568	0.146	0.235	0.136	0.17
R. Arm—Adipose Tissue (kg)	0.227	0.063	0.247	0.012 *	0.095	0.44	0.231	0.019 *
R. Arm—Soft Tissues (kg)	0.246	0.043 *	0.023	0.819	0.165	0.178	0.107	0.28
Trunk—Adipose Tissue (kg)	0.255	0.036 *	0.229	0.019 *	0.155	0.208	0.217	0.027 *
Trunk—Soft Tissues (kg)	0.199	0.104	0.098	0.322	0.06	0.624	0.152	0.123
L. Leg—Adipose Tissue (kg)	0.255	0.036 *	0.228	0.02 *	0.173	0.158	0.209	0.033 *
L. Leg—Soft Tissues (kg)	0.104	0.398	0.078	0.43	0.017	0.892	0.129	0.193
R. Leg—Adipose Tissue (kg)	0.267	0.028 *	0.228	0.02 *	0.143	0.246	0.211	0.031 *
R. Leg—Soft Tissues(kg)	0.238	0.051	0.085	0.391	0.119	0.335	0.125	0.205
B.M.R. (kcal)	0.14	0.255	0.042	0.673	0.055	0.655	0.14	0.157
T.E.E. (kcal)	0.137	0.267	−0.016	0.874	0.069	0.579	0.146	0.14
A.M.B. (years)	0.194	0.113	0.181	0.065	0.127	0.302	0.105	0.288
Impedance (Ω)	−0.066	0.593	−0.059	0.55	−0.044	0.723	0.01	0.919
Aim to Control (kg)	0.244	0.045 *	0.228	0.02 *	0.146	0.233	0.226	0.021 *
Duration of Therapy (weeks)	0.241	0.048 *	0.223	0.023 *	0.148	0.229	0.199	0.043 *

rho—Spearman’s correlation coefficient, L.B.M.—lean body mass (kg); S.L.M.—soft lean mass (kg); T.B.W.—total body water (%); P.B.F.—percent of body fat (%); B.M.I.—Body Mass Index (kg/m^2^); V.F.A.—visceral fat area (cm^2^); A.C.—abdominal circumference (cm); W.H.R.—waist hip ratio; Std. Mc.—standard body mass (kg); B.M.R.—basal metabolic rate (kcal); T.E.E.—total energy expenditure (kcal); A.M.B.—age matched of body (years); * *p* < 0.05.

**Table 5:** Correlation of body composition with physical functioning (P.F.) and physical restrictions (P.R.) domains in women from the study and control groups.

Parameters	Physical Functioning (P.F.)				Role Limitations due to Physical Problems (R.P.)			
	Study Group		Control Group		Study Group		Control Group	
	Correlation Coefficient	*p*	Correlation Coefficient	*p*	Correlation Coefficient	*p*	Correlation Coefficient	*p*
Body Mass (kg)	−0.244	0.05	−0.085	0.403	−0.292	0.016 *	−0.024	0.808
L.B.M. (kg)	−0.147	0.242	−0.006	0.952	−0.222	0.068	−0.038	0.703
Fat Tissue Mass (kg)	−0.224	0.073	−0.141	0.163	−0.289	0.017 *	0.024	0.811
S.L.M. (kg)	−0.123	0.329	0.01	0.919	−0.202	0.099	−0.032	0.752
Minerals (kg)	−0.235	0.06	−0.107	0.289	−0.298	0.014 *	−0.012	0.901
Protein (kg)	−0.069	0.585	0.069	0.497	−0.145	0.239	−0.036	0.718
T.B.W. (kg)	−0.211	0.091	−0.004	0.97	−0.253	0.037 *	−0.036	0.719
P.B.F. (%)	−0.144	0.253	−0.161	0.11	−0.244	0.045 *	0.076	0.446
B.M.I. (kg/m^2^)	−0.203	0.104	−0.14	0.165	−0.271	0.026 *	0.064	0.525
Fatness (%)	−0.33	0.08			−0.177	0.359		
Level (steps)	−0.133	0.293	−0.168	0.095	−0.242	0.047 *	0.067	0.506
V.F.A. (cm^2^)	−0.162	0.196	−0.176	0.08	−0.257	0.035 *	0.066	0.508
A.C. (cm)	−0.223	0.074	−0.146	0.148	−0.288	0.017 *	0.024	0.809
W.H.R.	−0.178	0.157	−0.177	0.079	−0.262	0.031 *	0.078	0.437
Std. Mc. (kg)	−0.031	0.809	0.167	0.1	0.025	0.841	−0.073	0.473
L. Arm—Adipose Tissue (kg)	−0.195	0.119	−0.144	0.154	−0.257	0.034 *	0.029	0.771
L. Arm—Soft Tissues (kg)	−0.246	0.048 *	0.019	0.854	−0.223	0.067	−0.04	0.692
R. Arm—Adipose Tissue (kg)	−0.158	0.209	−0.149	0.139	−0.209	0.087	0.025	0.805
R. Arm—Soft Tissues (kg)	−0.203	0.104	0.036	0.719	−0.278	0.022 *	−0.04	0.693
Trunk—Adipose Tissue (kg)	−0.226	0.071	−0.139	0.168	−0.299	0.013 *	0.026	0.794
Trunk—Soft Tissues (kg)	−0.108	0.393	−0.006	0.95	−0.171	0.162	−0.044	0.66
L. Leg—Adipose Tissue (kg)	−0.218	0.081	−0.141	0.162	−0.308	0.011 *	0.024	0.809
L. Leg—Soft Tissues (kg)	−0.059	0.641	0.009	0.932	−0.125	0.311	−0.035	0.729
R. Leg—Adipose Tissue (kg)	−0.223	0.074	−0.135	0.179	−0.287	0.018 *	0.033	0.742
R. Leg—Soft Tissues(kg)	−0.168	0.182	0.018	0.858	−0.246	0.043 *	−0.025	0.807
B.M.R. (kcal)	−0.037	0.771	0.042	0.681	−0.114	0.353	−0.052	0.606
T.E.E. (kcal)	−0.035	0.78	0.043	0.671	−0.102	0.409	−0.083	0.408
A.M.B. (years)	−0.346	0.005 **	−0.233	0.02 *	−0.298	0.014 *	0.036	0.716
Impedance (Ω)	0.095	0.452	0.013	0.9	0.19	0.121	−0.055	0.581
Aim to Control (kg)	−0.212	0.09	−0.142	0.158	−0.297	0.014 *	0.027	0.787
Duration of Therapy (weeks)	−0.215	0.085	−0.161	0.11	−0.297	0.014 *	0.023	0.816

rho—Spearman’s correlation coefficient, L.B.M.—lean body mass (kg); S.L.M.—soft lean mass (kg); T.B.W.—total body water (%); P.B.F.—per cent of body fat (%); B.M.I.—Body Mass Index (kg/m^2^); V.F.A.—visceral fat area (cm^2^); A.C.—abdominal circumference (cm); W.H.R.—waist hip ratio; Std. Mc.—standard body mass (kg); B.M.R.—basal metabolic rate (kcal); T.E.E.—total energy expenditure (kcal); A.M.B.—age matched of body (years); ** *p* < 0.01; * *p* < 0.05.

**Table 6:** Correlation between body mass composition and bodily pain (B.P.) and mental health (M.H.) domains in women from the study and control groups.

Parameters	Bodily Pain (B.L.)				Mental Health (M.H.)			
	Study Group		Control Group		Study Group		Control Group	
	Correlation Coefficient	*p*	Correlation Coefficient	*p*	Correlation Coefficient	*p*	Correlation Coefficient	*p*
Body Mass (kg)	−0.281	0.021 *	−0.115	0.251	−0.268	0.04 *	−0.085	0.394
L.B.M. (kg)	−0.249	0.043 *	−0.066	0.508	−0.176	0.183	−0.109	0.276
Fat Tissue Mass (kg)	−0.235	0.056	−0.127	0.204	−0.23	0.079	−0.106	0.287
S.L.M. (kg)	−0.246	0.044 *	−0.05	0.614	−0.172	0.193	−0.102	0.308
Minerals (kg)	−0.278	0.023 *	−0.124	0.214	−0.259	0.048 *	−0.118	0.236
Protein (kg)	−0.205	0.097	−0.005	0.96	−0.116	0.38	−0.067	0.501
T.B.W. (kg)	−0.281	0.021 *	−0.064	0.524	−0.202	0.125	−0.108	0.278
P.B.F. (%)	−0.132	0.289	−0.107	0.284	−0.186	0.157	−0.063	0.53
B.M.I. (kg/m^2^)	−0.271	0.027 *	−0.084	0.403	−0.215	0.102	−0.055	0.581
Fatness (%)	−0.229	0.232			−0.2	0.318		
Level (steps)	−0.135	0.277	−0.115	0.248	−0.186	0.158	−0.071	0.477
V.F.A. (cm^2^)	−0.138	0.265	−0.122	0.222	−0.183	0.166	−0.072	0.469
A.C. (cm)	−0.234	0.056	−0.132	0.186	−0.229	0.081	−0.108	0.279
W.H.R.	−0.136	0.273	−0.124	0.215	−0.19	0.149	−0.084	0.399
Std. Mc. (kg)	0.057	0.647	0.041	0.685	0	0.999	−0.071	0.482
L. Arm—Adipose Tissue (kg)	−0.229	0.063	−0.125	0.212	−0.174	0.188	−0.096	0.335
L. Arm—Soft Tissues (kg)	−0.233	0.058	−0.038	0.703	−0.163	0.216	−0.121	0.225
R. Arm—Adipose Tissue (kg)	−0.219	0.075	−0.133	0.181	−0.165	0.212	−0.096	0.335
R. Arm—Soft Tissues (kg)	−0.258	0.035 *	−0.034	0.737	−0.226	0.086	−0.119	0.233
Trunk—Adipose Tissue (kg)	−0.238	0.052	−0.127	0.204	−0.239	0.069	−0.106	0.29
Trunk—Soft Tissues (kg)	−0.189	0.126	−0.053	0.594	−0.16	0.227	−0.089	0.374
L. Leg—Adipose Tissue (kg)	−0.213	0.084	−0.128	0.198	−0.258	0.048 *	−0.101	0.312
L. Leg—Soft Tissues (kg)	−0.231	0.06	−0.028	0.78	−0.082	0.537	−0.089	0.374
R. Leg—Adipose Tissue (kg)	−0.238	0.053	−0.116	0.247	−0.222	0.091	−0.103	0.301
R. Leg—Soft Tissues(kg)	−0.28	0.022 *	−0.048	0.635	−0.211	0.109	−0.107	0.285
B.M.R. (kcal)	−0.168	0.175	−0.045	0.65	−0.135	0.309	−0.116	0.246
T.E.E. (kcal)	−0.106	0.392	−0.058	0.562	−0.157	0.234	−0.146	0.144
A.M.B. (years)	−0.186	0.131	−0.098	0.325	−0.108	0.414	−0.023	0.815
Impedance (Ω)	0.283	0.02 *	−0.095	0.341	0.115	0.387	−0.08	0.427
Aim to Control (kg)	−0.246	0.045 *	−0.142	0.155	−0.244	0.062	−0.086	0.389
Duration of Therapy (weeks)	−0.248	0.043 *	−0.128	0.2	−0.244	0.063	−0.074	0.46

rho—Spearman’s correlation coefficient, L.B.M.—lean body mass (kg); S.L.M.—Soft Lean Mass (kg); T.B.W.—Total Body Water (%); P.B.F.—percent of body fat (%); B.M.I.—Body Mass Index (kg/m^2^); V.F.A.—visceral fat area (cm^2^); A.C.—abdominal circumference (cm); W.H.R.—waist hip ratio; Std. Mc.—standard body mass (kg); B.M.R.—basal metabolic rate (kcal); T.E.E.—total energy expenditure (kcal); A.M.B.—age matched of body (years); * *p* < 0.05.

**Table 7:** Correlation between body composition and physical component summary domain (P.C.S.) in surveyed women.

Parameters	Physical Component Summary (P.C.S.)			
	Study Group		Control Group	
	Correlation Coefficient	*p*	Correlation Coefficient	*p*
Body Mass (kg)	−0.283	0.026 *	−0.147	0.146
L.B.M. (kg)	−0.193	0.132	−0.097	0.338
Fat Tissue Mass (kg)	−0.27	0.034 *	−0.184	0.067
S.L.M. (kg)	−0.18	0.16	−0.08	0.427
Minerals (kg)	−0.28	0.028 *	−0.168	0.095
Protein (kg)	−0.116	0.369	−0.027	0.788
T.B.W. (kg)	−0.257	0.044 *	−0.094	0.35
P.B.F. (%)	−0.2	0.12	−0.163	0.105
B.M.I. (kg/m^2^)	−0.247	0.053	−0.144	0.153
Fatness (%)	−0.274	0.151	***	***
Level (steps)	−0.184	0.153	−0.168	0.095
V.F.A. (cm^2^)	−0.209	0.104	−0.176	0.081
A.C. (cm)	−0.27	0.034 *	−0.189	0.059
W.H.R.	−0.217	0.09	−0.184	0.067
Std. Mc. (kg)	0.001	0.997	0.05	0.627
L. Arm—Adipose Tissue (kg)	−0.233	0.068	−0.177	0.079
L. Arm—Soft Tissues (kg)	−0.254	0.047 *	−0.071	0.483
R. Arm—Adipose Tissue (kg)	−0.197	0.125	−0.189	0.059
R. Arm—Soft Tissues (kg)	−0.252	0.048 *	−0.056	0.578
Trunk—Adipose Tissue (kg)	−0.278	0.029 *	−0.185	0.066
Trunk—Soft Tissues (kg)	−0.153	0.234	−0.095	0.347
L. Leg—Adipose Tissue (kg)	−0.275	0.031 *	−0.181	0.071
L. Leg—Soft Tissues (kg)	−0.15	0.246	−0.074	0.462
R. Leg—Adipose Tissue (kg)	−0.268	0.035 *	−0.181	0.071
R. Leg—Soft Tissues(kg)	−0.219	0.087	−0.061	0.548
B.M.R. (kcal)	−0.128	0.321	−0.067	0.51
T.E.E. (kcal)	−0.087	0.504	−0.008	0.939
A.M.B. (years)	−0.253	0.047 *	−0.162	0.106
Impedance (Ω)	0.119	0.355	−0.034	0.735
Aim to Control (kg)	−0.271	0.033 *	−0.165	0.1
Duration of Therapy (weeks)	−0.274	0.031 *	−0.188	0.061

rho—Spearman’s correlation coefficient, L.B.M.—lean body mass (kg); S.L.M.—soft lean mass (kg); T.B.W.—total body water (%); P.B.F.—percent of body fat (%); B.M.I.—Body Mass Index (kg/m^2^); V.F.A.—visceral fat area (cm^2^); A.C.—abdominal circumference (cm); W.H.R.—waist hip ratio; Std. Mc.—standard body mass (kg); B.M.R.—basal metabolic rate (kcal); T.E.E.—total energy expenditure (kcal); A.M.B.—age matched of body (years); * *p* < 0.05.
